# Practical Uranalysis and Urinary Diagnosis

**Published:** 1894-08

**Authors:** 


Practical Uranalysis and Urinary Diagnosis.

A manual for the use of practitioners and students, with numerous illustrations, including colored photo-engravings. By Charles W. Purdy, M. D., of Chicago, author of *Bright's Disease and Allied Affections of the Kidneys, Dia-*

*betes: Its Causes, Symptoms, and Treatment*, etc. A one-volume practical and systematic work of about 350 crown-octavo pages, in two parts, subdivided into twelve sections, and an appendix. This important new book is just announced by the well-known house of The F. A. Davis Company, 1914 and 1916 Cherry Street, Philadelphia, who will issue the work in September, 1894. The book will be first-class in quality of paper, presswork, and binding, and the price most reasonable, namely, $2.50, net, in extra cloth.

